# Perceived threat of Alzheimer’s disease and dementia predicting health behavior

**DOI:** 10.1002/alz.093029

**Published:** 2025-01-09

**Authors:** Ravneet K. Dhaliwal, Claudia Jacova, Hannah Marx, Frank Robertson, Jacklyn Gehling

**Affiliations:** ^1^ Pacific University, Hillsboro, OR USA

## Abstract

**Background:**

Factors influencing health behaviors among older adults experiencing cognitive changes are important to understand in order to successfully promote preventative public health programs for Alzheimer’s disease and related dementias (ADRD). The Health Belief Model (HBM) has been utilized to investigate and predict health behaviors based on an individuals’ perceptions and beliefs. The aim of our study was to investigate the contribution of perceived threat (risk and worry) to individuals seeking care for cognitive changes.

**Method:**

Participants of this study were 279 adults (M age = 73.8, range 60‐96; 54% female, 89% white, M education (years) = 14.9) recruited from the community, without neurocognitive diagnoses, and residing in the United States. They completed an online survey through Qualtrics. Participants rated their risk for developing ADRD and their worry about developing ADRD on 5‐point scales (risk: 1 = much lower than average to 5 = much higher than average; worry: 1 = not at all worried to 5 = very worried). They also answered questions about whether they noticed difficulties with their memory and cognition (yes/no), and whether they had seen a doctor for these changes (yes/no).

**Result:**

Following the HBM, perceived threat is separated into two variables: perceived susceptibility (risk) averaged M = 2.48 (58.4% rating in the 3‐5 range) and perceived severity (worry) averaged M = 2.05 (25.8% rating in the 3‐5 range). There were 101 participants (36%) who endorsed perceiving difficulties with their memory or cognition. Of these, only 3 participants (M age = 70.3, range 70‐71; 2 females, M education (years) = 14.7) said ‘yes’ to seeing their doctor about memory or cognitive difficulties (3%), preempting further predictive analyses.

**Conclusion:**

We found that despite endorsing memory/cognitive difficulties, and reporting above average risk and worry about ADRD, an exceedingly small number of adults aged ≥60 had consulted their doctor. Our findings align with previous research reporting that medical help‐seeking does not keep apace with perceived susceptibility to ADRD and worry in older adults. As a result, our understanding of factors that influence health behaviors, such as seeking care early on, remains limited.

